# Supplementary material for: Caveolin-1-Mediated Tumor Suppression Is Linked to Reduced HIF1α S-Nitrosylation and Transcriptional Activity in Hypoxia
Source: Cancers (Basel). 2020 Aug 20;12(9):2349. doi: 10.3390/cancers12092349 (PMC7565942; doi:10.3390/cancers12092349)
Supplement: Supplementary file 1 [file cancers-12-02349-s001.docx]

Supplementary Materials: Caveolin-1-Mediated Tumor Suppression Is Linked to Reduced HIF1α S-Nitrosylation and Transcriptional Activity in Hypoxia


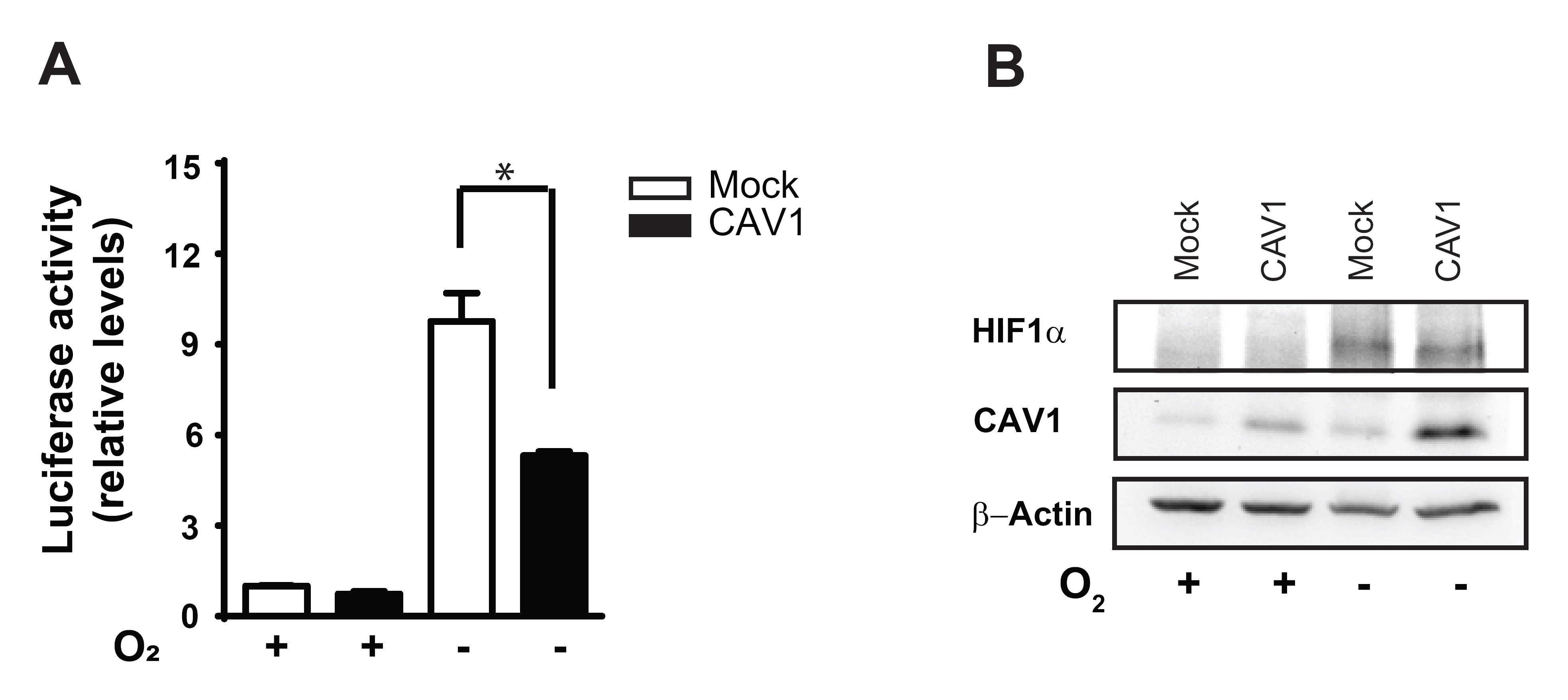


**Figure S1.** Inhibition of HIF1-gene activation by caveolin-1 expression during hypoxia in HEK293T cells. **(A)** HEK293T cells (1x10^6^) were transiently cotransfected using the calcium-phosphate method with 1.3 µg of CAV-1 encoding plasmid pLacIOP(CAV-1) or empty vector pLacIOP, pGL3-HRE together with the pON plasmid. After induction of CAV1 expression with IPTG 1mM for 24h, the cells were incubated in hypoxia (-, 1% O_2_ ) or normoxia (+, 20% O_2_) for an additional 24 h period. Then, cells were subsequently lysed and HIF1-gene reporter activity was determined as described in the methodology section. **B)** Changes in HIF1α protein levels were determined by Western blotting. HEK293T were transfected as detailed in A). After 24 h of hypoxia, cell extracts were obtained and HIF1α, CAV-1, and β-actin (loading control) protein levels were determined by Western blotting. In both cases, data are expressed as an means ± SEM of results from n = 3 independent experiments (*p<0.05 Mock vs CAV-1 in hypoxia).


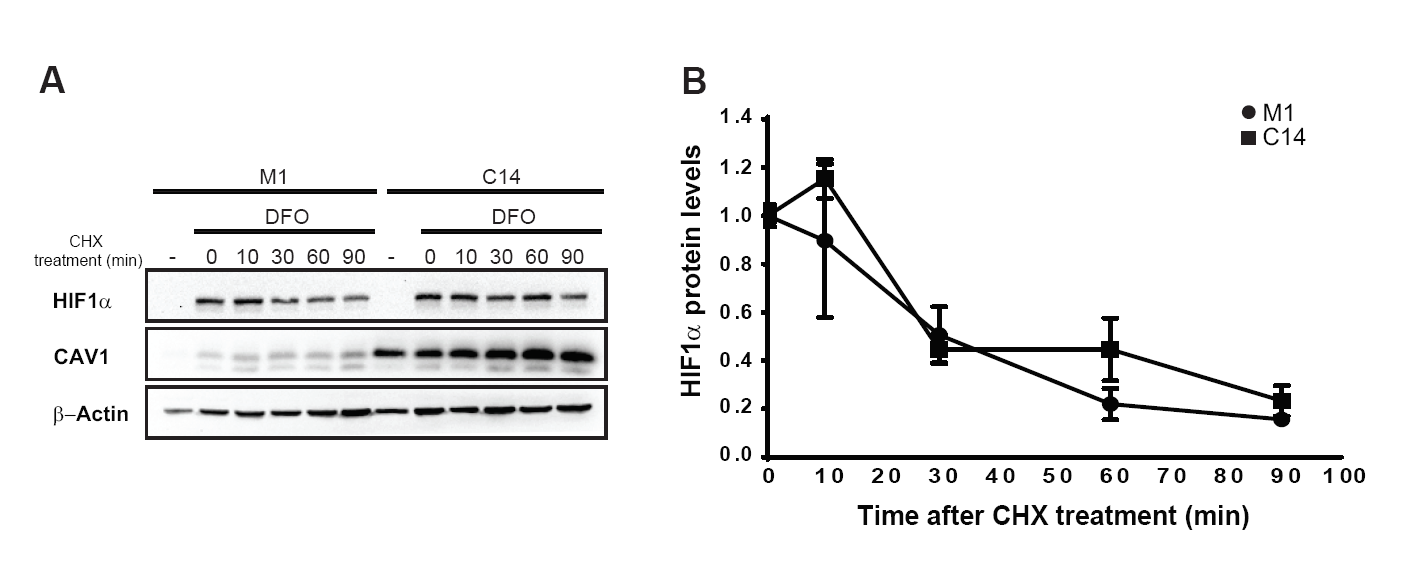


**Figure S2.** Analysis of HIF1α half-life. HT29(US) (clone M1 and C14) cells were treated with DFO (200 µM, 18 h). Protein synthesis was blocked using CHX (100 µg/mL) for 0 to 90 min and HIF1α protein levels were evaluated by Western blotting. Normalized HIF1α protein levels were adjusted to a first-order decay equation and HIF1α half-life was calculated by adjusting normalized densitometric data to a first-order decay equation using GraphPad Prism 5 Sofware. The half-life was calculated from the equation T1/2 = -0,693/k used to fit data sets, where “k” is the first order decay constant.


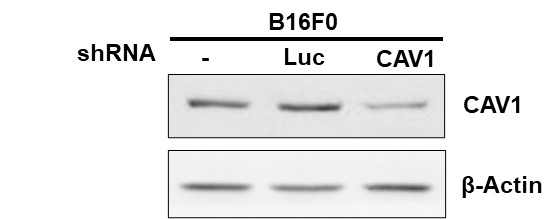


**Figure S3.** ShRNA-mediated silencing of Cav-1 protein in B16F10 cells. CAV-1 protein levels in B16F0 (shLuc) and shCAV-1 cells. B16F0 cells stably transfected with an shRNA targeting CAV-1 (shCAV-1) or Luciferase (shLuc, control) [1], were cultured until cells reached 70% confluency. After that, the cells were lysed, and CAV-1 and actin (loading control) were analyzed in cell extracts by Western blotting.

**Reference:**

1. Urra, H.; Torres, V.A.; Ortiz, R.J.; Lobos, L.; Diaz, M.I.; Diaz, N.; Hartel, S.; Leyton, L.; Quest, A.F. Caveolin-1-enhanced motility and focal adhesion turnover require tyrosine-14 but not accumulation to the rear in metastatic cancer cells. *PLoS One* **2012**, *7*, e33085, doi:10.1371/journal.pone.0033085 PONE-D-11-06157 [pii].

Western Blots:

**Figure 1.** Qualitative blot.

|  |
| --- |
| **Figure 3. (A)** |
|  |
| **Figure 3. (B)** |
|  |
|  |

**Figure 3. (D)** Qualitative blot.

**Figure 4.** (**C**) Qualitative blot.

**Figure S1.** Qualitative blot.

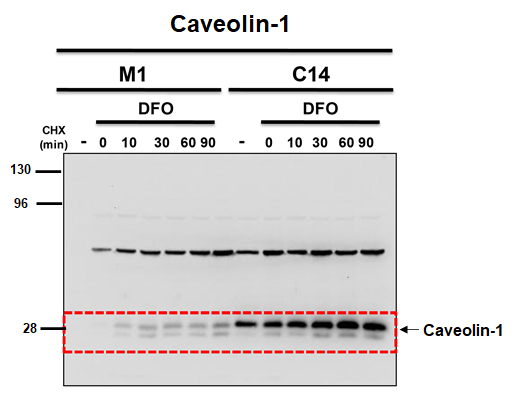


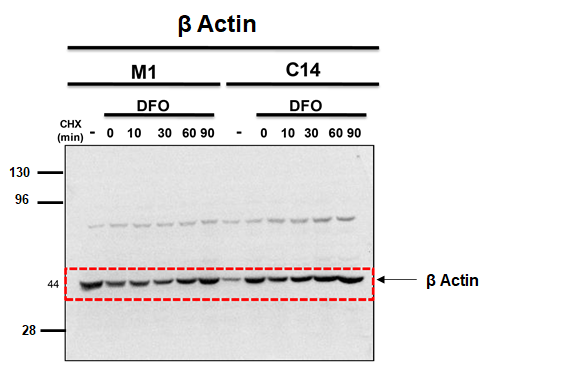


**Figure S2.** Qualitative blot.


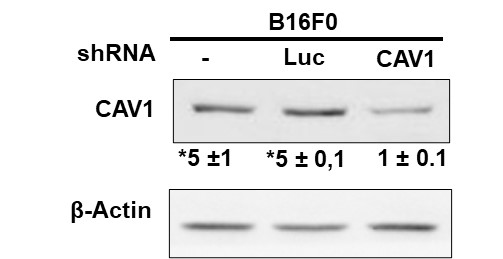


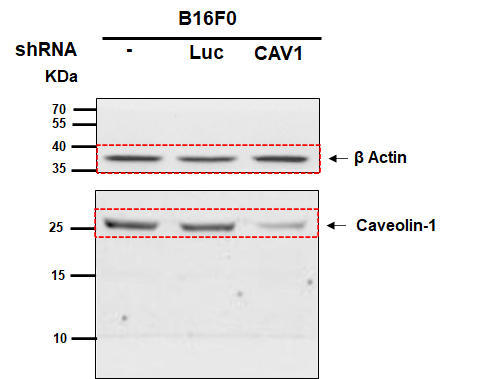


**Figure S3.** Qualitative blot.
